# Clinical implications of CT-detected ascites in gastric cancer: association with peritoneal metastasis and systemic inflammatory response

**DOI:** 10.1186/s13244-024-01818-1

**Published:** 2024-10-07

**Authors:** Mengying Xu, Dan Liu, Le Wang, Shuangshuang Sun, Song Liu, Zhengyang Zhou

**Affiliations:** 1 Department of Radiology, Nanjing Drum Tower Hospital, Affiliated Hospital of Medical School, Nanjing University, 210008 Nanjing, China; 2 Department of Radiology, Nanjing Drum Tower Hospital, Nanjing Drum Tower Hospital Clinical College of Nanjing Medical University, 210008 Nanjing, China

**Keywords:** Stomach neoplasms, Ascites, Peritoneal metastasis, Systemic inflammatory indexes, Tomography (x-ray computed)

## Abstract

**Objectives:**

This study aimed to evaluate the diagnostic significance of computed tomography (CT) detected ascites in gastric cancer (GC) with peritoneal metastasis (PM) and investigate its association with systemic inflammatory response.

**Methods:**

This retrospective study included 111 GCs with ascites (PM: *n* = 51; No PM: *n* = 60). Systemic inflammatory indexes, tumor markers, and the CT-assessed characteristics of ascites were collected. The differences in parameters between the two groups were analyzed. Diagnostic performance was obtained by receiver operating characteristic curve analysis. The association between the volume of ascites and clinical characteristics was evaluated with correlation analysis.

**Results:**

In this study, over half of GCs with ascites were not involved with PM. The systemic immune-inflammation index (SII), neutrophil-lymphocyte ratio (NLR), platelet-lymphocyte ratio (PLR), five tumor markers, and the characteristics of ascites showed significant differences between the two groups (all *p* < 0.05). Among them, SII, NLR, PLR, and the volume of ascites achieved the areas under the curve of 0.700, 0.698, 0.704, and 0.903, respectively. Moreover, the volumes of ascites showed positive correlations with SII, NLR, and PLR in GCs with PM, and the volumes of ascites detected in the upper abdomen were more strongly correlated with CA125 level (all *p* < 0.05).

**Conclusion:**

Many GCs with CT-detected ascites did not occur with synchronous PM. The presence of upper abdominal ascites had certain clinical significance for diagnosing PM in GCs. Systemic inflammatory indexes were elevated and positively correlated with the volume of ascites in GCs with PM, which might suggest the enhanced systemic inflammatory response.

**Critical relevance statement:**

CT-detected ascites in the upper abdomen played an indicative role in identifying synchronous PM in GCs, and the systemic inflammatory response was enhanced in GCs with PM, which might be helpful for clinical evaluation.

**Key Points:**

- Many GCs with CT-detected ascites did not occur with synchronous PM.
- CT-detected ascites in the upper abdomen help in identifying PM in GCs.
- GCs with PM showed elevated systemic inflammatory indexes and enhanced systemic inflammatory response.

**Graphical Abstract:**

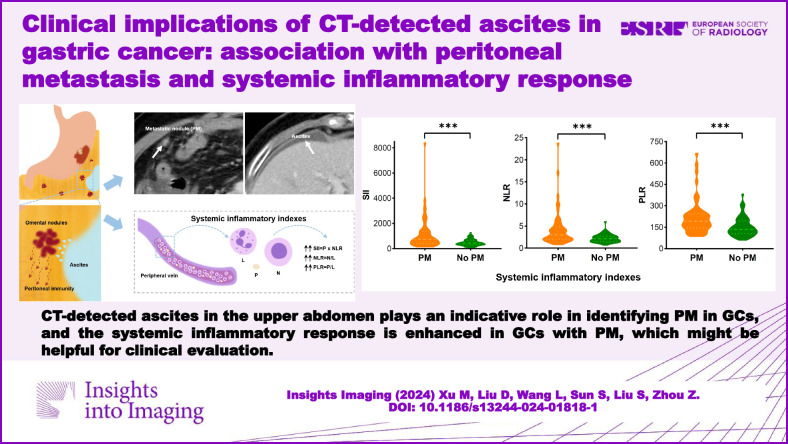

## Introduction

Gastric cancer (GC) is the fifth most common cancer and one of the leading causes of cancer-related mortalities globally [1]. Many patients with GC presented with advanced stages when initially diagnosed, resulting in poor prognosis [2–4]. Peritoneal metastasis (PM) occurs in more than 50% of GCs with distant metastasis and remains the most threatening metastatic mode of GC-related death [5–7].

Malignant ascites, accounting for approximately 10% of all cases of ascites, is defined as the result of abnormal accumulation of fluid within the peritoneal cavity in patients with advanced malignant tumors and is also known as the common clinical complication of GCs with peritoneal dissemination [8, 9]. In addition, previous studies also indicated that peritoneal carcinomatosis was identified as a clinically important risk factor for malignant ascite development [10, 11]. In clinical practice, some GCs without PM confirmed by surgery could also be detected with ascites on preoperative computed tomography (CT) images. Some previous studies analyzed the relationship between CT-detected ascites and PM in GCs [12, 13]. Yajima et al reported that CT-detected ascites suggested the presence of PM [12]. While Chang et al indicated that CT-detected small amount of ascites in GCs could not necessarily be identified as PM [13]. Moreover, previous clinical studies assessing ascites in GCs, including the grading system and quantitative analysis, mainly focused on the efficacy and prognosis evaluation in GCs with PM [14–17]. The differences in the distribution and volume of ascites between GCs with peritoneal dissemination and without peritoneal involvement have not been well-studied. Therefore, a detailed analysis of GCs with ascites may be helpful for clinical identification.

Systemic inflammatory response is defined as the entity composed of circulating cytokines, circulating immune cells, small inflammatory proteins, and acute-phase proteins [18]. Moreover, the magnitude of the systemic inflammatory response can be reflected with clinically easily available peripheral blood laboratory tests, including neutrophil-lymphocyte ratio (NLR), platelet-lymphocyte ratio (PLR), and systemic immune-inflammation index (SII) calculated by lymphocyte, neutrophil, and platelet counts [19]. In recent years, the important role of systemic inflammatory response in the cancer-related survival of malignant tumors has been investigated and recognized increasingly [20–26]. Mungan et al demonstrated that the NLR and PLR could predict mortality and morbidity in GCs after gastrectomy [26]. Moreover, Weng et al showed that the values of NLR and PLR increased in ovarian cancer patients accompanied by malignant ascites, indicating enhanced systemic inflammation [27]. SII is a joint tool and a novel systemic inflammatory biomarker. Yan et al reported that SII was recommended as the factor for clinical treatment decision-making in colorectal cancers accompanied by peritoneal carcinomatosis [25]. However, the changes in systemic inflammatory response in GCs with peritoneal dissemination and its correlation with ascites have not been well explored up to now.

In this study, we first aimed to investigate the clinical significance of CT-detected ascites in GCs with peritoneal dissemination. Then, we aimed to explore the changes in systemic inflammatory response in GCs with peritoneal dissemination and to evaluate its association with ascites.

## Materials and methods

### Patients

**Fig. 1:**
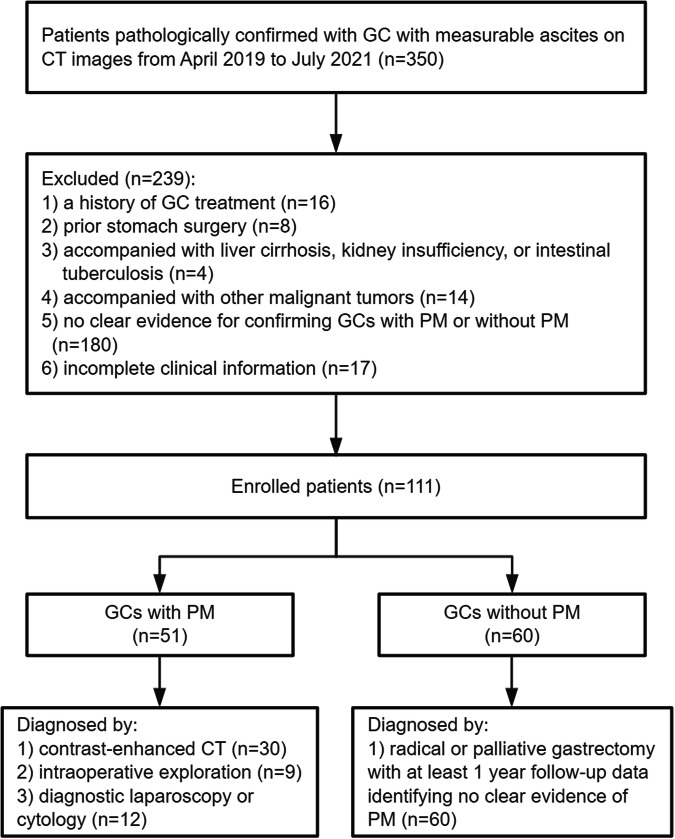
Flowchart of the patient selection in this study. GC, gastric cancer; CT, computed tomography; PM, peritoneal metastasis

This retrospective study followed the principles of the Declaration of Helsinki and was approved by the Institutional Review Board of our institution. Because of the retrospective nature of this study, the requirement for obtaining informed consent was waived by the ethics committee. From April 2019 to July 2021, 350 cases of GC with ascites admitted to our hospital were consecutively enrolled in this study by searching the electronic image system. Patients were included in the analysis with the following criteria: (1) pathologically confirmed with GC by endoscopic biopsy; (2) qualified abdominal contrast-enhanced CT images with the presence of measurable ascites on initial admission. Patients were excluded with any of the following criteria: (1) a history of GC treatment (*n* = 16); (2) prior stomach surgery (*n* = 8); (3) accompanied with liver cirrhosis, kidney insufficiency, or intestinal tuberculosis (*n* = 4); (4) accompanied with other malignant tumors (*n* = 14); (5) no clear evidence for confirming GCs with PM or without PM (*n* = 180); (6) incomplete clinical information (*n* = 17). In this study, GCs with synchronous PM were diagnosed by CT images, intraoperative exploration, diagnostic laparoscopy, or cytology. Meanwhile, GCs without PM were confirmed by surgery and needed at least 1-year follow-up data, including imaging and clinical follow-up records indicating no clear evidence of PM. The flowchart with detailed patient selection is shown in Fig. 1.

Finally, this study included 111 cases of GC with ascites (male, 61; female, 50; median age, 58 years; age range, 24–86 years). In detail, GCs with ascites were divided into two groups (GCs with PM: *n* = 51; GCs without PM: *n* = 60). Table 1 summarizes the demographic and clinical characteristics of all patients.

**Table 1:** Demographic data and clinical information of all patients

Characteristics	All patients (*n* = 111)	Peritoneal metastasis (*n* = 51)	No peritoneal metastasis (*n* = 60)	*p*
Demographic data				
Gender				0.255
Male	61 (55.0%)	31 (60.8%)	30 (50.0%)	
Female	50 (45.0%)	20 (39.2%)	30 (50.0%)	
Age (years)				0.893
< 60	58 (52.3%)	27 (52.9%)	31 (51.7%)	
≥ 60	53 (47.7%)	24 (47.1%)	29 (48.3%)	
Systemic inflammatory indexes				
SII	498.67 (322.64, 836.00)	732.00 (401.62, 1377.50)	434.28 (306.31, 665.50)	< 0.001*
NLR	2.33 (1.73, 3.50)	3.00 (2.00, 4.83)	2.16 (1.47, 2.74)	< 0.001*
PLR	157.14 (116.67, 221.82)	191.43 (144.29, 242.94)	134.72 (105.44, 192.97)	< 0.001*
Tumor markers				
CEA (ng/mL)	1.34 (0.55, 3.92)	1.84 (0.68, 7.89)	1.16 (0.50, 2.08)	0.033*
AFP (ng/mL)	1.80 (1.30, 3.40)	2.10 (1.30, 4.40)	1.65 (1.30, 3.08)	0.169
CA242 (U/mL)	5.13 (2.58, 11.10)	6.35 (2.75, 55.06)	4.76 (2.54, 7.54)	0.012*
CA199 (U/mL)	11.21 (5.37, 26.94)	20.02 (7.21, 186.70)	8.50 (5.33, 15.82)	0.001*
CA125 (U/mL)	12.90 (6.50, 38.70)	38.70 (17.50, 78.30)	7.90 (4.30, 11.03)	< 0.001*
CA724 (U/mL)	2.26 (1.50, 13.34)	9.84 (2.26, 67.83)	1.50 (1.27, 2.73)	< 0.001*

Quantitative variables are presented as median (interquartile range)

*SII* systemic immune-inflammation index, *NLR* neutrophil-lymphocyte ratio, *PLR* platelet-lymphocyte ratio, *CEA* carcinoembryonic antigen, *AFP* alpha-fetoprotein, *CA* carbohydrate antigen

* *p * <  0.05 with chi-square test or Fisher’s exact test (*n* <  5) in categorical variables and with Mann-Whitney *U*-test in quantitative variables

### Demographic and laboratory data collection

Clinical information, including gender, age, serum carcinoembryonic antigen (CEA), alpha-fetoprotein, carbohydrate antigen (CA) 125, CA199, CA242, CA724, the absolute lymphocyte, neutrophil, and platelet counts, were collected from the electronic medical records within two weeks of the CT examination. Then, derived NLR values were calculated with the absolute neutrophil and lymphocyte counts. PLR values were calculated with the absolute platelet and lymphocyte counts. The values of SII were determined as platelet count × NLR.

### CT image acquisition

A 64-row scanner (uCT 780, United Imaging, Shanghai, China) was performed for abdominal contrast-enhanced CT examination. All patients were informed to fast for no less than 6 h and drink 600–1000 mL of warm water orally before image acquisition. The CT examination covered the entire abdomen. The acquisition settings of the scanner were as follows: tube voltage of 120 kV, tube current of 150–250 mA, field of view 35–50 cm, matrix of 512 × 512, rotation time of 0.7 s, and pitch of 1.0875. After the unenhanced scan, contrast-enhanced CT images were obtained 40 s (arterial phase), 70 s (venous phase), and 180 s (delayed phase) delay after the infusion of 1.5 mL/kg of iodinated contrast agent (Omnipaque 350 mg I/mL, GE Healthcare) via the antecubital vein at a rate of 3 mL/s by a high-pressure syringe [28].

### Ascites assessment and volume of interest segmentation

#### Assessment of ascites distribution

The CT images of each patient were evaluated by one junior radiologist (Reader 1, with four years of abdominal diagnosis experience) at first and finally confirmed by one senior radiologist (Reader 2, with twelve years of abdominal diagnosis experience) without informing any clinical information [29–31]. Then, the distribution of ascites was determined according to the location as follows: type A was defined as ascites not detected in the upper abdomen; type B was defined as ascites detected in the upper abdomen.

#### Assessment of ascites grade

The assessment of ascites grade was also performed according to the above process. Considering that each patient enrolled in this study analysis was accompanied by CT-detected ascites, the grade of ascites was determined as follows [16, 17]: grade 1 was defined as ascites identified only in the upper abdominal or pelvic cavity; grade 2 was defined as ascites identified neither grade 1 nor grade 3; grade 3 was defined as ascites extending continuously from the upper abdominal cavity to the pelvic cavity.

#### Measurement of ascites volume

Venous phase CT images of all patients were loaded into an open-source software (3D Slicer, version 5.2.2; http://slicer.org) for measurement [32]. A previous study has shown that the volume of ascites measured on CT was significantly correlated with the amount of ascites found on autopsy [33]. Then, the volumes of interest (VOIs) were manually segmented along the boundaries of ascites, avoiding abdominal organs and other tissues by the junior radiologist. After the confirmation of segmentation by the senior radiologist, the segmentation maps were created with consensus. The upper abdominal and lower abdominal pelvic regions were divided based on the boundary lines of the costal arch inferior margin and anterior superior iliac spine. Subsequently, the volumes of ascites of each patient, including the upper abdominal, lower abdominal pelvic, and the entire abdominopelvic cavities, were calculated and recorded.

### Statistical analysis

The differences between GCs with synchronous PM and without PM in categorical variables, including demographic data, the distribution of ascites, and the grade of ascites, were assessed by the chi-square or Fisher’s exact test (*n* < 5). The differences in quantitative variables were analyzed utilizing the Mann-Whitney *U-*test after the normality testing of the Shapiro-Wilk test. Receiver operating characteristic (ROC) curves analysis with generated optimal cutoff values were utilized to evaluate the diagnostic performance of quantitative variables. The association between the volume of ascites and clinical characteristics was estimated by Spearman’s rank correlation analysis. Data analysis was performed using SPSS v25.0 and MedCalc statistical software. Two-sided *p*-value < 0.05 was considered statistically significant.

## Results

### Clinical information

#### Patient characteristics

**Fig. 2:**
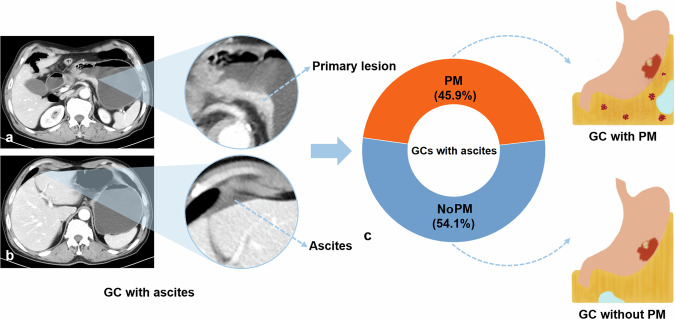
Proportion of peritoneal metastasis (PM) in gastric cancers (GCs) with ascites and corresponding illustrations. **a**, **b** Computed tomography (CT) images of a sixty-eight-year-old male pathologically diagnosed as GC (dotted blue arrow) with ascites (dotted blue arrow) detected on CT scan. The patient was confirmed without PM by surgery and follow-up data. **c** The pie chart shows the proportion of PM in GCs with ascites in this study. The orange area of the pie chart reflects the proportion of GCs with PM (45.9%), and the blue area demonstrates the proportion of GCs without PM (54.1%). GC, gastric cancer; PM, peritoneal metastasis

Among the 111 GCs with ascites enrolled in the study analysis, there were 51 (45.9%) patients with synchronous PM and 60 (54.1%) patients without PM (Fig. 2). Within the 51 patients with synchronous PM, 30 (58.8%) cases were diagnosed by contrast-enhanced CT, 9 (17.6%) cases were confirmed by intraoperative exploration, and 12 (23.5%) cases were confirmed by diagnostic laparoscopy or cytology. Meanwhile, for GCs with PM diagnosed by intraoperative exploration, diagnostic laparoscopy, or cytology, the time interval between the above examinations and CT scans was within two weeks. In the remaining 60 patients with GC, all cases underwent radical or palliative gastrectomy in our hospital and were confirmed without synchronous PM. In addition, those 60 patients with GC had at least 1 year of follow-up data after surgery, indicating no clear evidence of PM. For demographic analysis, there were no significant differences in gender and age between the two groups (both *p* > 0.05).

#### Systemic inflammatory indexes

**Fig. 3:**
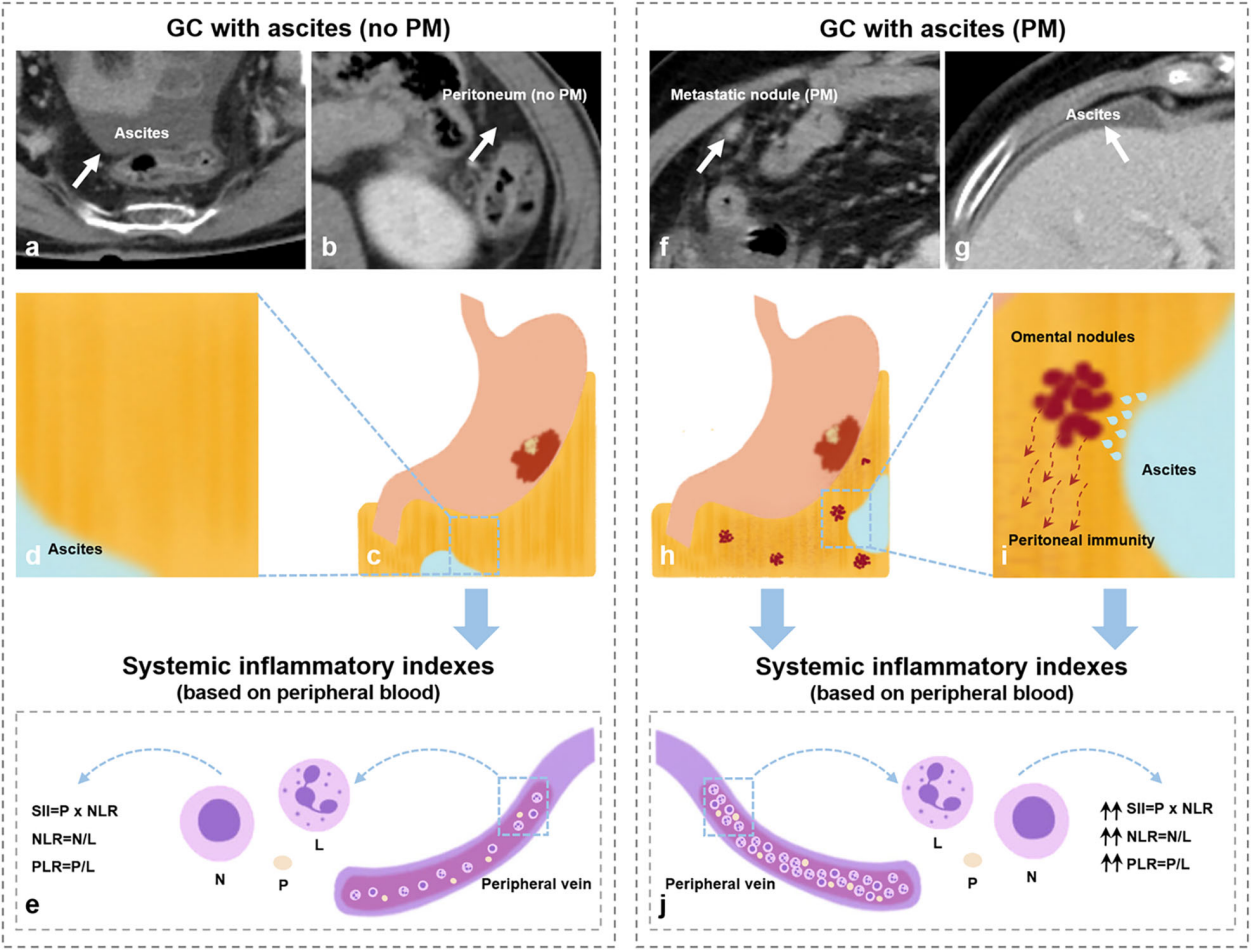
Representative illustrations and computed tomography (CT) images of gastric cancers (GCs) with ascites. **a**, **b** A case of GC with ascites confirmed without peritoneal metastasis (PM) by surgery and follow-up data. **a** The CT image shows a small amount of ascites (white arrow) detected in the pelvic cavity. **b** The CT image shows a clear appearance of the peritoneum (white arrow). **c** Illustration of GC with ascites (no PM). **d** A magnification of the blue box area on image **c**. **e** Illustration of the collection of systemic inflammatory indexes. Systemic inflammatory indexes, including systemic immune-inflammation index (SII), neutrophil-lymphocyte ratio (NLR), and platelet-lymphocyte ratio (PLR), were calculated by the absolute lymphocyte, neutrophil, and platelet counts from the peripheral venous blood. **f**, **g** A case of GC with ascites diagnosed with PM by contrast-enhanced CT. **f** The CT image shows an enhanced nodule (white arrow) in the right abdominal cavity. **g** The CT image shows a small amount of perihepatic ascites. **h** Illustration of GC with ascites (PM). **i** A magnification of the blue box area on image **h** presents the stimulation of metastatic nodules to the adjacent omentum, which leads to the formation of ascites and peritoneal immunity [39]. **j** The illustration indicates significantly elevated SII, NLR, and PLR levels in GCs with PM. GC, gastric cancer; PM, peritoneal metastasis; SII, systemic immune-inflammation index; NLR, neutrophil-lymphocyte ratio; PLR, platelet-lymphocyte ratio; N, the neutrophil count; L, the lymphocyte count; P, the platelet count

**Fig. 4:**
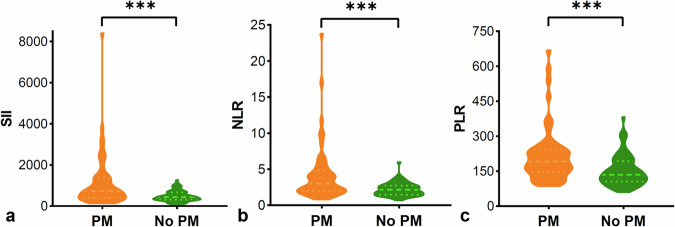
The violin plot in the distribution of systemic inflammatory indexes between gastric cancers (GCs) with peritoneal metastasis (PM) and without PM. **a**–**c** The systemic immune-inflammation index (SII), neutrophil-lymphocyte ratio (NLR), and platelet-lymphocyte ratio (PLR) showed significant differences between the two groups. PM, peritoneal metastasis; SII, systemic immune-inflammation index; NLR, neutrophil-lymphocyte ratio; PLR, platelet-lymphocyte ratio

**Fig. 5:**
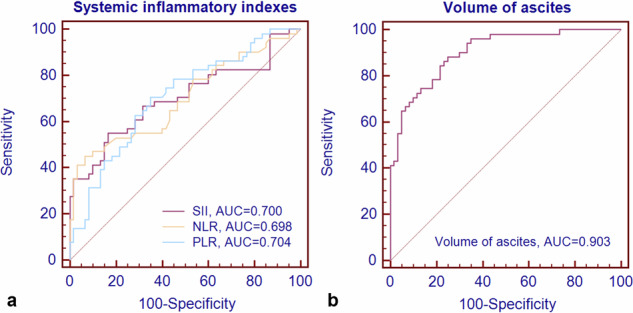
Receiver operating characteristic curves of the systemic inflammatory indexes and the volume of ascites for discriminating between gastric cancers (GCs) with peritoneal metastasis (PM) and without PM. **a** The systemic immune-inflammation index (SII), neutrophil-lymphocyte ratio (NLR), and platelet-lymphocyte ratio (PLR) achieved the areas under the curve (AUCs) of 0.700, 0.698, and 0.704, respectively. **b** The volume of ascites achieved the AUC of 0.903. AUC, area under the curve; SII, systemic immune-inflammation index; NLR, neutrophil-lymphocyte ratio; PLR, platelet-lymphocyte ratio

The univariate analysis for systemic inflammatory indexes between synchronous PM and without PM is listed in Table 1. Systemic inflammatory indexes, including NLR, PLR, and SII, were significantly higher in the synchronous PM group than in GCs without PM (all *p* < 0.001, Figs. 3 and 4). The corresponding optimum cutoff values were 3.5, 154.2, and 708.0, respectively. The ROC curves of the above three inflammatory markers are shown in Fig. 5, with achieved areas under the curve (AUCs) of 0.698, 0.704, and 0.700, respectively.

#### Tumor markers

The univariate analysis for tumor markers between synchronous PM and without PM is shown in Table 1. Patients with synchronous PM showed significantly higher levels of CA125, CA724, CEA, CA242, and CA199 (all *p* < 0.05). The corresponding optimum cutoff values were 11.7 U/mL, 3.3 U/mL, 2.8 ng/mL, 16.0 U/mL, and 26.9 U/mL, which achieved the AUC values of 0.902, 0.808, 0.617, 0.638, and 0.680, respectively (Fig. S1).

### Ascites assessment and volume of interest segmentation

#### The distribution of ascites

The distribution of ascites showed a significant difference between the two groups (*p* < 0.001, Table 2). Type A and type B ascites were detected in patients with synchronous PM, and type B (33, 64.7%) accounted for a higher proportion. For GCs without PM, type A of ascites (53, 88.3%) occupied a higher proportion. In addition, among the 40 patients with ascites also detected in the upper abdomen, 33 (82.5%) patients confirmed with synchronous PM.

**Table 2:** Univariate analysis of CT characteristics of all patients

Characteristics	Peritoneal metastasis (*n* = 51)	No peritoneal metastasis (*n* = 60)	*p*
Distribution of ascites			< 0.001*
Type A	18 (35.3%)	53 (88.3%)	
Type B	33 (64.7%)	7 (11.7%)	
Grade of ascites [16, 17]			< 0.001*
Grade 1	18 (35.3%)	52 (86.7%)	
Grade 2	23 (45.1%)	8 (13.3%)	
Grade 3	10 (19.6%)	0 (0.0%)	
Volume of ascites (cm^3^)	23.40 (7.95, 163.10)	2.82 (1.42, 5.70)	< 0.001*

Type A was defined as ascites not detected in the upper abdomen; Type B was defined as ascites detected in the upper abdomen; Grade 1 was defined as ascites identified only in the upper abdominal or pelvic cavity; Grade 2 was defined as ascites identified neither grade 1 nor grade 3; Grade 3 was defined as ascites extending continuously from the upper abdominal cavity to the pelvic cavity [16, 17]. Quantitative variables are presented as median (interquartile range)

*CT* computed tomography

* *p* < 0.05 with chi-square test or Fisher’s exact test (*n* <  5) in categorical variables and with Mann-Whitney *U*-test in quantitative variables

#### The grade of ascites

The grade of ascites also demonstrated a significant difference between the two groups (*p* < 0.001, Table 2). Grades 1–3 of ascites were found in patients with synchronous PM, and grade 2 of ascites (23, 45.1%) accounted for the highest proportion. For GCs without PM, grade 1 of ascites (52, 86.7%) occupied the highest proportion, while grade 3 of ascites was not detected. In addition, ascites were detected in the pelvic cavity of all patients by both radiologists in this study.

#### The volume of ascites

The segmentation maps of ascites annotated by the radiologists were utilized to calculate the volume of ascites. Then, the total volumes of ascites of all patients were obtained, which showed significant differences between the two groups (*p* < 0.001, Table 2). The generated AUC value was 0.903, with the optimum cutoff value of 5.6 mL (Fig. 5).

### Correlation between the volume of ascites and clinical characteristics

#### Ascites volume associated with systemic inflammatory indexes

For GCs with synchronous PM, the volumes of ascites showed positive correlations with NLR, PLR, and SII (*r* = 0.321, 0.450, and 0.318, respectively, all *p* < 0.05). However, for GCs without PM, the volumes of ascites indicated no correlations with NLR, PLR, or SII (all *p* > 0.05). The detailed results are listed in Table 3.

**Table 3:** Correlation between the volume of ascites and the level of systemic inflammatory indexes

Characteristics	Volume of ascites			
	Peritoneal metastasis		No peritoneal metastasis	
	*r*	*p*	*r*	*p*
Systemic inflammatory indexes				
SII	0.318	0.023*	0.158	0.227
NLR	0.321	0.022*	0.104	0.428
PLR	0.450	0.001*	0.241	0.064

*SII* systemic immune-inflammation index, *NLR* neutrophil-lymphocyte ratio, *PLR* platelet-lymphocyte ratio, *r* Spearman’s rank correlation coefficient

* *p*  <  0.05 with Spearman’s rank correlation analysis

#### Distribution of ascites associated with tumor markers

Univariate analysis showed that five tumor markers differed significantly between GCs with synchronous PM and without PM. Positive correlations were found between the values of CA125 and the volumes of ascites located in different regions, including the upper abdominal, lower abdominal pelvic, and the entire abdominopelvic cavities (*r* = 0.668, 0.603, and 0.642, respectively, all *p* < 0.001). The values of CA724 also correlated positively with the volumes of ascites located in different regions (*r* = 0.561, 0.333, and 0.375, respectively, all *p* < 0.001). The values of CA199 correlated positively with the volumes of ascites located in the upper abdominal cavity (*r* = 0.375, *p* = 0.017). The detailed results are shown in Table 4.

**Table 4:** Correlation between the level of tumor markers and the volume of ascites

Characteristics	Volume of ascites					
	Abdominopelvic cavity		Upper abdominal cavity		Lower abdominal pelvic cavity	
	*r*	*p*	*r*	*p*	*r*	*p*
Tumor markers						
CA125	0.642	< 0.001*	0.668	< 0.001*	0.603	< 0.001*
CA724	0.375	< 0.001*	0.561	< 0.001*	0.333	< 0.001*
CEA	0.074	0.441	0.164	0.312	0.049	0.608
CA242	0.083	0.385	0.208	0.198	0.042	0.660
CA199	0.144	0.131	0.375	0.017*	0.094	0.327

*CEA* carcinoembryonic antigen, *CA* carbohydrate antigen, *r* Spearman’s rank correlation coefficient

* *p* <  0.05 with Spearman’s rank correlation analysis

## Discussion

It has been indicated that CT-detected small amount of ascites in GCs could not necessarily be identified as PM [13]. GCs with peritoneal dissemination and the presence of ascites were reported with extremely poor survival compared with patients without ascites or the involvement of PM. Moreover, the detection of ascites was an independent risk factor for overall survival and progression-free survival [34]. Although ascites is one of the complications of PM, many patients without the involvement of PM are accompanied by ascites at initial diagnosis. Therefore, the clinical significance of CT-detected ascites in GCs needs further analysis.

In this study, more than half of GCs accompanied by ascites were not involved with synchronous PM, indicating that the presence of ascites may not necessarily be identified as PM in GCs. As for the distribution of ascites, 40 (36.0%) patients had ascites presented in the upper abdomen, among which the patients accompanied with synchronous PM (33, 82.5%) accounted for much higher frequency. Therefore, the presence of ascites in the upper abdomen may be more valuable for the diagnosis of PM. Our results agreed with the previous study that suggested the detection of perihepatic and upper abdominal ascites indicating PM strongly [35]. Moreover, a recent review also indicated that the left and right upper abdomen quadrants were the most common areas of laparoscopic exploration and were also the main regions of peritoneal fluid aspiration in GCs, which emphasized the importance of ascites in the upper abdominal region for PM [36].

Many previous studies on GCs with PM evaluated the distribution and determined the grade of ascites for analysis [16, 17, 37]. Part of the studies utilized the five-point method for the quantitative measurement of ascites. However, the method was not suitable for patients with small amount of ascites [14, 15, 38]. In addition to evaluating the distribution of ascites, our study also segmented the VOIs of ascites and further calculated their volumes presented in different positions. Our results demonstrated that the volume of ascites differed significantly between the two groups and showed satisfactory diagnostic performance for PM (AUC = 0.903).

The peritoneal immunity and immune microenvironment in the peritoneal cavity were reported to be markedly changed when GCs with peritoneal dissemination [39–42]. In this study, we found that the level of systemic inflammatory markers, including NLR, PLR, and SII, increased significantly and were positively correlated with the volumes of ascites in GCs with synchronous PM. However, the above inflammatory indexes showed no correlations with the volumes of ascites in GCs without PM. It suggested that the systemic inflammatory response was enhanced in GCs with peritoneal involvement, and those clinically accessible indexes might serve as indicative biomarkers for PM and efficacy evaluation in clinical practice.

In recent years, the clinical importance of tumor markers in the diagnosis, efficacy, and prognosis evaluation of GCs has been indicated increasingly [43–47]. Emoto et al reported that the elevated serum CA125 level could reflect the progression of PM and was correlated with the quantity of ascites [43]. Compared with the volumes of ascites in abdominopelvic and lower abdominal pelvic cavities, our study manifested that the volumes of ascites in the upper abdomen were more strongly correlated with the CA125 level, which further suggested the indicative role of ascites detected in the upper abdomen.

Our study had some limitations. First, considering the difficulty in diagnosing the status of “no metastases” in patients without visible PM, the follow-up evaluation was added to this study. Therefore, the relatively rigorous patient selection process further improved the status of “no metastases” and could make this study more rigorous, though it might lead to sample selection bias. Second, our study focused on the clinical implications of CT-detected ascites in GCs in clinical practice, which was different from previous studies for predicting PM in GCs. Therefore, this study mainly analyzed the CT characteristics of ascites other than the features of the primary tumors. Third, our work retrospectively evaluated the link between GCs with PM, ascites, and systemic inflammatory response. Whether there is a cascade effect between them needs further studies in the future.

In conclusion, the presence of upper abdominal ascites had certain clinical significance in the diagnosis of PM in GCs. Systemic inflammatory indexes were elevated and positively correlated with the volume of ascites in GCs with PM, which might suggest the enhanced systemic inflammatory response.

## Supplementary information

ELECTRONIC SUPPLEMENTARY MATERIAL

## Data Availability

The datasets used and analyzed during the current study are available from the corresponding author upon reasonable request.

## Abbreviations

AUC: Area under the curve
CA: Carbohydrate antigen
CEA: Serum carcinoembryonic antigen
CT: Computed tomography
GC: Gastric cancer
NLR: Neutrophil-lymphocyte ratio
PLR: Platelet-lymphocyte ratio
PM: Peritoneal metastasis
ROC: Receiver operating characteristic
SII: Systemic immune-inflammation index
VOI: Volume of interest
