# Supplementary material for: Clinical implications of CT-detected ascites in gastric cancer: association with peritoneal metastasis and systemic inflammatory response
Source: Insights Imaging. 2024 Oct 7;15:237. doi: 10.1186/s13244-024-01818-1 (PMC11460829; doi:10.1186/s13244-024-01818-1)
Supplement: Supplementary file 1 — ELECTRONIC SUPPLEMENTARY MATERIAL [file 13244_2024_1818_MOESM1_ESM.pdf]

Clinical implications of CT-detected ascites in gastric cancer: association with peritoneal metastasis and systemic inflammatory response

ELECTRONIC SUPPLEMENTARY MATERIAL

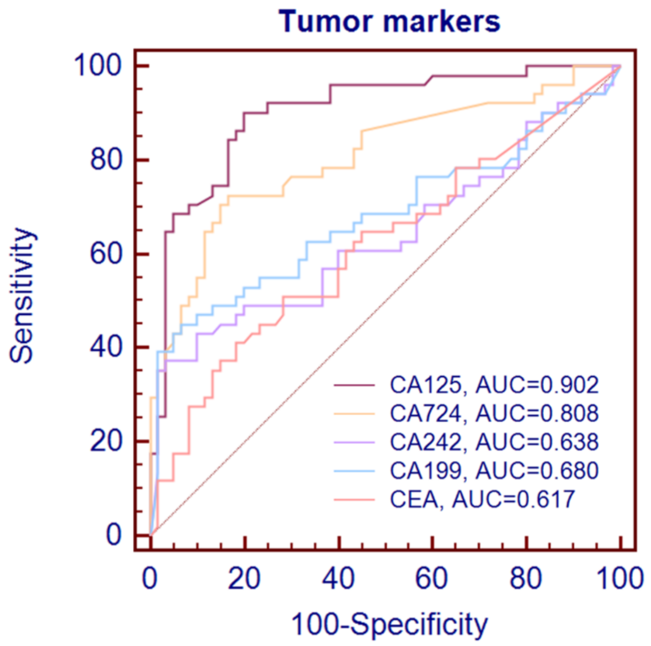

Fig. S1 Receiver operating characteristic curves of tumor markers for discriminating between gastric cancers (GCs) with peritoneal metastasis (PM) and without PM. AUC, area under the curve; CA, carbohydrate antigen; CEA, serum carcinoembryonic antigen.
